# Magnetic Particle Imaging: Current and Future Applications, Magnetic Nanoparticle Synthesis Methods and Safety Measures

**DOI:** 10.3390/ijms22147651

**Published:** 2021-07-17

**Authors:** Caroline Billings, Mitchell Langley, Gavin Warrington, Farzin Mashali, Jacqueline Anne Johnson

**Affiliations:** 1 College of Veterinary Medicine, University of Tennessee, Knoxville, TN 37996, USA; cbilli10@vols.utk.edu; 2 Department of Mechanical, Aerospace and Biomedical Engineering, University of Tennessee, Knoxville, TN 37996, USA; mlangle5@vols.utk.edu (M.L.); gwarring@vols.utk.edu (G.W.); fmashali@vols.utk.edu (F.M.); 3 Department of Mechanical, Aerospace and Biomedical Engineering, University of Tennessee Space Institute, Tullahoma, TN 37388, USA

**Keywords:** magnetic nanoparticles, magnetic particle imaging, superparamagnetic iron oxide, nanoparticle safety

## Abstract

Magnetic nanoparticles (MNPs) have a wide range of applications; an area of particular interest is magnetic particle imaging (MPI). MPI is an imaging modality that utilizes superparamagnetic iron oxide particles (SPIONs) as tracer particles to produce highly sensitive and specific images in a broad range of applications, including cardiovascular, neuroimaging, tumor imaging, magnetic hyperthermia and cellular tracking. While there are hurdles to overcome, including accessibility of products, and an understanding of safety and toxicity profiles, MPI has the potential to revolutionize research and clinical biomedical imaging. This review will explore a brief history of MPI, MNP synthesis methods, current and future applications, and safety concerns associated with this newly emerging imaging modality.

## 1. Introduction

### 1.1. Background and Significance

It is undeniable that the fields of science and medicine are advancing daily. As preventative medicine and therapeutics advance, there is a necessity for increased sensitivity and precision in diagnostic and therapeutic procedures. In the past one to two decades [1], a promising field has emerged, described by Sun et al. [2] as a major class of nanoscale materials with the potential to revolutionize current clinical diagnostic and therapeutic techniques. This is the field of magnetic nanoparticles (MNPs). In this review, we will emphasize the role that MNPs play within magnetic particle imaging (MPI), as MPI has emerged as a promising non-invasive imaging technique [3].

MPI is a tracer-based, functional and tomographic imaging modality, which determines the spatial distribution of MNPs [4]. MNPs are a widely diverse group of materials that have relevant and potentially ground-breaking applications in a wide variety of biomedical arenas including drug delivery, cell targeting, magnetic hyperthermia, and diagnostic imaging [5]. MPI was first conceived in 2001 by Gleich et al., first publicly presented in 2005 as a method with potential use for rapid vascular and small intestinal imaging [6], and had initial commercial products available in 2013 and 2014 [1,7,8]. Surrounding these milestones exists an incredible amount of research and development, including the utilization of superparamagnetic iron oxide nanoparticle (SPION) imaging agents such as Ferucarbotran (Resovist^®^, Bayer Healthcare) and Feridex^®^ (Ferumoxides, Berlex Laboratories), which are most often used as contrast agents for magnetic resonance imaging (MRI) [2] as MPI tracer agents, and the development of MPI scanners appropriate for rodents [7,9]. Without argument, there is an immense amount of research being pursued within the field of MPI. In fact, since the first public presentation on MPI, there have been annual meetings devoted solely to the presentation and discussion of further investigations into synthesis methods for magnetic nanoparticles, magnetic particle imaging systems and hardware, as well as applications and limitations [4] of MPI. Again without argument, much more research and development is required to allow MPI to reach its full potential and appropriate use for clinical imaging.

### 1.2. Principles and Methods

As stated above, MPI is a tracer-based, functional, and tomographic imaging modality, which determines the spatial distribution of MNPs [4]. MPI can be utilized in conjunction with other imaging techniques such as MRI or computed tomography (CT), or alone as an MPI system utilizing a special MPI scanner [2,10]. MPI scanner technologies appropriate for people are not currently available for clinical use, although there are groups that have working models, including a small animal scanner [6,8,11].

SPIONs have emerged as the most utilized MNP for MPI applications because of their superparamagnetism. Superparamagnetism is an important component to MPI, as utilization of SPIONs allows for high-order harmonics of excitation frequencies once an oscillating magnetic field is applied. This facilitates the key features of MPI, which include quantitative mapping of MNPs with high spatial and temporal resolution [12]. Additionally, SPIONs are stable and easy to prepare, they offer intrinsic biocompatibility [13], and are relatively inexpensive [14,15]. SPIONs are also amenable to modification of functionalization through surface coatings, which can affect cellular uptake of nanoparticles [16], biodistribution [17], blood circulation [18] and metabolism of nanoparticles [19]. With these characteristics in mind, the rise of SPIONs as a crucial element in MPI is clear. 

MPI relies on tracking the tracer materials of SPIONs to form highly sensitive and quantitative 3D images via direct measurement of SPION tracers present within a particular area [8]. This is performed with high spatial and temporal resolution [8,12,20,21], and MPI signals obtained solely from particles, not surrounding tissues [10]. This phenomena of MPI signals being obtained from particles instead of surrounding tissues is in part a result of the diamagnetic properties of organs and tissues. These diamagnetic properties cause organs and tissues to appear transparent on MPI scans [11,12,22,23]. MPI is fast and derives its signal from the non-linear re-magnetization response of SPIONs to an oscillating magnetic field [12].

In general terms, the signal production and acquisition in MPI occurs via the application of an external magnetic field, which aligns magnetic moments of SPIONs to create a net magnetization vector. Magnetic behavior of SPIONs can best be explained by utilizing the Langevin theory, assuming that the SPIONs are in a state of constant thermal equilibrium [8,12]. Within the Langevin theory, it is assumed that particles are constantly moving and possess randomly aligned magnetic moments that lead to a net magnetization of zero. With the application of an external magnetic field, magnetic moments of the particles will align and result in a net magnetization vector. This relationship between the applied magnetic field and resultant magnetization will be non-linear. The magnetization will have a steep climb early on and will reach saturation at a particular field strength. Once this point is achieved, most particles will be in alignment with the magnetic field and the magnetization response will be unchanged. This is described as a fixed magnetization response and is critical for spatial encoding and generation of MPI images [6,8,12]. If time-varying fields are applied, particle magnetization responses will be delayed for a quantifiable and known amount of time. This is referred to as a relaxation time [6,12]. At this point, particles may undergo either Brownian relaxation, which is a physical particle rotation, or Néel relaxation, which is an internal magnetic moment rotation [7,12,24]. It is possible for a combination of both rotations to occur within a fluid environment and with dependency on the applied frequency [12]. These described SPION relaxation properties and behaviors in the presence of an applied external magnetic field serve as the basis for MPI signal generation and acquisition.

Fundamentals of SPION properties in MPI signal generation and acquisition are described above. There are of course, multiple components to MPI systems, and now the basics of scanner systems will be discussed. MPI relies on the application of an oscillating magnetic field, which is called the drive field. This field typically has amplitudes between 0.1 and 20 mT and will alter the magnetization of SPIONs utilizing transmit coils [6,8,12]. Magnetic flux density will be evaluated to determine the change in magnetization of the nanoparticles. This is performed by measuring the voltage induced by using appropriate receiver coils [8,12]. Determination of locations of MNPs for spatial encoding, which is a highlight feature of MPI, is possible through application of an additional static magnetic field gradient. This gradient will typically have a strength of approximately 4 T/m and will be superimposed onto the drive field to establish a field-free point (FFP) within the volume of interest [6,8,11,12]. With that FFP established, particles within the FFP will be able to follow the excitation field and form the signal within the receiving coils [8,12]. The non-linear relationship between the applied magnetic field and the SPIONs leads to the harmonics in the detected signal [12,22], which can then be filtered to isolate the higher-order frequencies from the received signal [6,12]. Harmonics can then be manipulated with a Fourier transformation to allow quantification of the local concentration of SPIONs [12].

After MPI signals are generated and acquired, they must be converted into images. This is performed by utilizing reconstruction algorithms, typically either harmonic-space MPI or x-space MPI [12,23]. Harmonic-space MPI was first applied by Gleich and Weizenecker [6,12], and the measured signal is dependent upon particle concentration and system function [12,25], where system function describes the relationship between the acquired MPI signal and the spatial origin [12,26]. X-space MPI was developed by Conolly et al. and expresses the MPI images as a convolution of the spatial distributions of the SPIONs with the point spread function of the system [12].

### 1.3. Benefits

Magnetic particle imaging is an innovative imaging modality that is being developed to add strength and diversity to current imaging techniques such as MRI, CT, bioluminescence imaging (BLI), positron emission tomography (PET), and single photon emission computed tomography (SPECT) [12,27]. While each of these imaging techniques possess clinical utility, they are also accompanied by shortcomings. For example, MRI is a commonly utilized and powerful diagnostic tool in many clinical arenas, including cancer and cardiovascular imaging [28]. Recently, the value has come into question, as limitations to MRI are recognized. Limitations include long scan times, lack of standards for quantitative measures for clinical usage, and inefficient recognition of false positive results prior to initiating treatment [29]. Additionally, the various imaging techniques present a level of risk to patients, including radiation exposure during nuclear imaging procedures such as PET and CT scans [30], and gadolinium exposure during MRI, which can lead to systemic accumulation or nephrogenic systemic fibrosis in certain patients [31]. With these limitations in mind, the desire for a safe, efficient, ultra-sensitive and specific, multipurpose imaging system that will produce quantifiable 3D images is obvious—and leads us to MPI.

Benefits of MPI include

1. Quantitative distribution mapping of administered magnetic nanoparticles with high spatial and temporal resolutions. This is produced by the changing magnetization of SPION tracers, causing occurrence of higher-order harmonics of the excitation frequency [11,12,23]. In fact, when compared to MRI, which can utilize similar agents to MPI, such as Resovist [9], the electronic superparamagnetism detected by MPI is 22 million-fold stronger than the nuclear MRI magnetization [32,33].

2. Creation of clear images by eliminating issues associated with background signals [10] through exploitation of diamagnetic properties of tissue and organs [11,12,23], as well as improved signal-to-noise ratios [32], thus eliminating a principal challenge of MRI.

3. Linear quantification of the amount and location of SPIONs regardless of tissue depth, [12,13], a major advantage over imaging modalities such as BLI, where signal penetration is an issue [20], and MRI, where direct quantification is impossible.

4. Diversity in potential applications, from early initial detection of a small abnormality [34] to specific diagnosis and monitoring of therapy and even utilization in image-guided magnetic hyperthermia treatment [13], is a major advantage of MPI. The potential to perform all these imaging tasks with a single modality, or two imaging modalities in combination instead of multiple in succession, especially one that does not expose patients to ionizing radiation [20], is a pivotal concept for modern medicine.

### 1.4. Challenges

Considering the described and perceived strengths of MNPs and MPI, the question arises regarding the lack of clinical use of this system. While there are many advantages to MPI and it undoubtedly has the potential to improve biomedical imaging, there are also many obstacles and challenges. These challenges include ideal MNP development, safety concerns and practical implementation [2,4,20,35,36].

Although SPIONs have emerged as a major player for use in MPI, it is still not a “one size fits all” scenario, and there are many variables that influence performance. These variables are present in every stage of development, including formulation of the iron oxide nanoparticle, synthesizing particles to control for magnetization [2], and obtaining appropriate signal sensitivity [36]. Additionally, while SPIONs are often referred to colloquially as being non-toxic in vitro [2], there is debate about whether iron oxide nanoparticles are inherently non-toxic. This stems from a question regarding the long-term fate of the magnetic nanoparticles and the products of MNP degradation [37]. This leads to a conversation of whether the iron oxide core of the MNP is ultimately broken down and incorporated naturally into newly formed erythrocytes [38] in a manner that avoids toxicity [37] or if further work is necessary to determine long-term metabolism and fate in vivo [2].

Additional safety concerns include the risks of peripheral nerve stimulation (PNS) and tissue heating [7]. PNS is a phenomenon attributed to magnetostimulation that causes sensory responses in muscle and is described by patients as a tingling or poking sensation [39]. PNS is noted as a limiting factor in MRI, as well as a potential limiting or risk factor in MPI [40]. Tissue heating is a risk when utilizing MPI for magnetic hyperthermia (MH), as hyperthermia has the inherent potential to be damaging to adjacent tissues [13]. Tissue heating is also a risk in general when time-varying magnetic fields are applied to the body [41]. This is apparent in MRI as well as MPI, although it is suggested that the homogenous drive field of MPI, which operates in the 5–25 kHz range, can create different electric field patterns than MRI. This variation in electric field patterns may lessen the risk of tissue heating [41] in MPI, particularly because tissue heating seems to become an issue above 25kHz frequency range [7].

Lastly, the practical implementation of MPI has proven challenging. Most currently available MPI systems are designed to accommodate rodents [7], and there are challenges in upscaling an MPI scanner that is appropriate in size and able to facilitate human imaging, although development of such a scanner is being actively pursued [11,12]. Additionally, on the topic of practical implementation, there are certainly situations where MPI alone is insufficient. These situations include the need for morphological description, which is typically not provided by tracer-based imaging modalities and requires overlay with a CT or MR image [8,42,43].

Ultimately, the minimal amount of research on the safety of MNPs combined with challenges developing a safe and appropriate human MPI scanner will prolong the time to available and commonplace clinical use. This provides a need for additional research to investigate performance and potential toxicity of MNPs alongside MPI scanner development. This review article provides a current understanding of MPI applications, challenges, and synthesis methods and safety concerns encompassing magnetic nanoparticles.

## 2. Magnetic Nanoparticle Synthesis Methods

As the utility of MNPs across various applications becomes more widely accepted, new methods for MNP synthesis are being developed. Specifically, as SPIONs hold such strong promise in the field of MPI, there are many synthesis methods reported. The most common methods include co-precipitation, thermal decomposition, hydrothermal synthesis, microemulsion, electrochemical synthesis and bacterial synthesis [44,45,46,47].

Presently, producing a colloidally stable batch of iron oxide nanoparticles is dependent on particle size, surface chemistry, density, and aqueous conditions [15,48]. Creating monodispersed iron oxide nanoparticles provides consistency, reproducible results, and predictable responses. This is critical, as limited reproducibility is a challenge in large-scale production and scalable production will be an important step to MPI becoming available for clinical use [37]. This is also important for applications such as MH, where high colloidal stability against precipitation and agglomeration of MNPs is required to maintain long-term heating efficiency [44]. Research has demonstrated that thermal decomposition techniques using nonpolar solvents produce highly monodispersed MNPs with specific surface coatings [15]. Additionally, a recent study showed that performing thermal decomposition in the presence of oleic acid synthesized highly monodispersed and stable 12.3 nm magnetite nanoparticles with a deviation of 1.0 nm [15]. These innovative techniques indicate that there are many useful synthesis methods that are able to produce uniform, effective nanoparticles. Of note is that while thermal decomposition methods utilizing nonpolar solvents produce desirable results, they are relatively high in price. The price can be lowered by pursuing polyol methods, but those protocols are currently inappropriate for scalable particle production [37].

Lastly, the importance of surface coatings has grown dramatically, as they are crucial for a variety of functions, including increased biocompatibility, the option for functionalization [47], and may also play a role in hyperthermia performance both in vitro and in vivo [37]. Kim et al. [15] applied surface coatings that included sodium dodecyl sulfate, cetyltrimethylammonium bromide, and polyethylene glycol (PEG). They demonstrated high colloidal stability of MNPs due to particle size and electrostatic and steric repulsive forces. As a result, the surface coatings prevented particle aggregation and membrane blocking [15], which can increase biocompatibility in vivo. This holds promise and opens areas for future work, as a technique for reproducible coating and purification is still necessary to achieve scalable production of MNPs [37].

## 3. MPI Applications

### 3.1. Cancer Imaging

Four of the most common oncology imaging modalities are CT, MRI, PET and SPECT. Each of these methods possess limitations regarding cancer imaging and detection. Sensitivity and resolution limit these methods from detecting smaller numbers of cells. Currently, these imaging methods can create a 3-D image of a tumor that has approximately 10^9^ cells [49]. The Gompertzian growth curve of a solid tumor indicates that tumor growth evolves from one cell and once there are approximately 10^5^ number of cells an angiogenic switch occurs that logarithmically increases the cell/tumor activity [50]. The goal of oncology imaging is to be able to detect tumor growth with the smallest number of cells possible for earlier diagnosis and intervention.

MPI research is pushing the boundaries of conventional oncology imaging, especially sensitivity and resolution, by utilizing the intrinsic characteristics of MNPs and SPIONS. Song et al. [34] explored this approach by creating Janus iron oxide particles coated with a semiconducting polymer. Those MNPs demonstrated 7-fold the intensity of commercial MRI tracers and 3-fold the intensity of commercial MPI tracers. The Janus MNPs properly imaged as few as 250 cancer cells in vivo. Compared to the clinical imaging capability of 10^9^ cancer cells, 250 cells indicate a substantial significant difference in resolution and sensitivity.

Yu et al. [33] explored the possibility of in vivo cancer MPI imaging in a xenograft breast tumor rat model utilizing long circulating SPIO tracer (LS-008). Their work relied on the enhanced permeability and retention (EPR) effect that is understood to allow nanoparticles to preferentially accumulate in tumors due to the leaky vasculature of the tumor [33,51]. They were able to demonstrate preferential accumulation of the particles within the tumors, with particles present within the tumor up to six days post injection, and a dose dependent increase in concentration of the tracer in blood. These findings provide confidence in the EPR effect and the quantitative abilities of MPI in vivo. This work led Yu et al. [33] to pose the question of MPI’s efficacy in less vascular tumors as well as metastatic tumors, given the heterogeneity of neoplasia, prior to suggesting clinical use of MPI for cancer imaging.

Israel et al. [52] focused a review on iron oxide nanoparticles for brain cancer imaging and eventual therapy. An emphasis was placed on the need for nanoparticles that are predictable and well controlled, and able to cross the blood–brain barrier (BBB), potentially through application of magnetic field, or through surface functionalization such as cyclic arginine-glycine-aspartate (cRGD) to target NPs into brain tumors, as there are exceptional limitations in current brain cancer imaging and therapies. Israel et al. [52] concluded that iron oxide nanoparticles are an area of promise, with the potential to image and treat brain tumors in the future, and acknowledged unresolved biosafety concerns, focusing on surface engineering and functionalities as an area to promote safety and biocompatibility, as surface engineering can mediate transport of NPs across the BBB through biologic pathways such as endocytosis or transcytosis [53].

**Figure 1:**
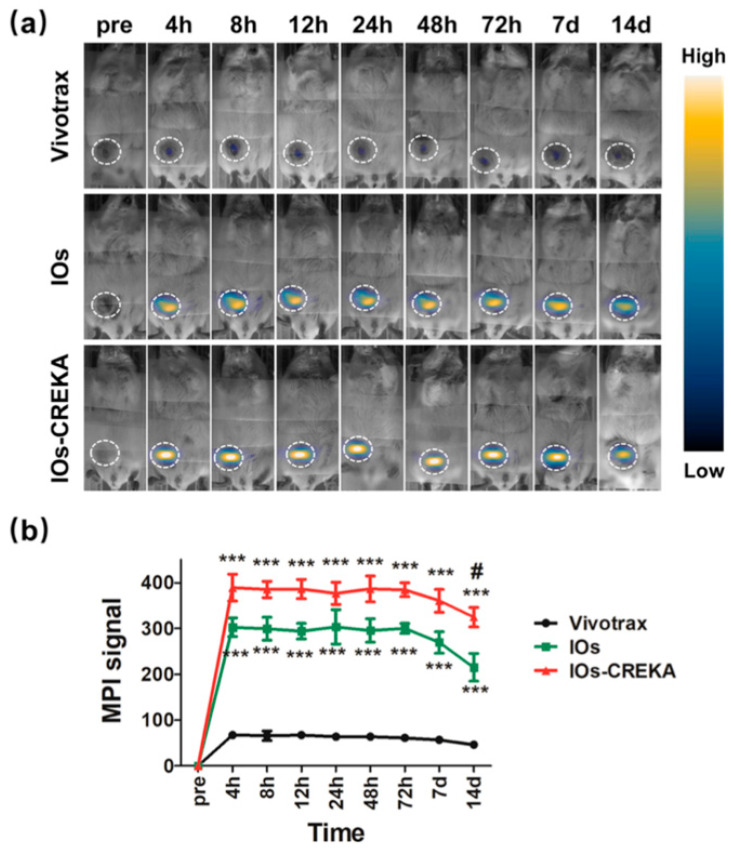
MRI**/**MPI image of nanoparticle distribution from a mouse model (**a**) which shows improvement of MPI signal intensity using CREKA peptide functionalization of iron oxide nanoparticles (**b**). *** *p* < 0.001, IOs-CREKA NPs or IOs compared with Vivotrax group; ^#^ *p* < 0.05, IOs versus IO-CREKA NPs.Reprinted with permission from Du et al. [54].

Another focus of MPI is enhancing the distribution of tracers within the tumor to create more sensitive images. Nanoparticles can be coated during synthesis to increase biocompatibility and facilitate functionalization [52]. Certain peptide sequences or antibody proteins can be added to the surface of MNPs to more accurately target cancer cells. Du et al. [54] used a tumor targeting agent called CREKA to functionalize PEG-coated iron oxide nanoparticles. CREKA is a penta-peptide known to bind to fibrin-fibronectin complexes expressed by cancer cells. MNPs were functionalized with CREKA to selectively accumulate within cancer cells after systemic administration of MNPs. Results, which are visualized in Figure 1, demonstrated an MPI signal 1.5-fold more intense than non-functionalized iron oxide nanoparticles and 5-fold more intense than Vivotrax which is commercially available.

Similarly, Arami et al. [55] functionalized PEG-coated iron oxide nanoparticles with lactoferrin. Lactoferrin was chosen for its particular sensitivity to C6 brain cancer cells. Nanoparticles were then administered intravenously in a murine brain cancer model. After two hours of circulation, MPI intensity in the brain was measured. Lactoferrin functionalized particles were 3-fold more intense than the non-functionalized iron oxide nanoparticles in vivo.

The studies of Arami, Du and Yu provide a glimpse into the multitude of possibilities regarding nanoparticle functionalization and usage to promote cancer detection and appropriate tumor distribution. Research is ongoing to determine ideal surface compositions for particular cancer cell affinity. Proper tumor distribution and optimization of magnetic nanoparticle signal responses will provide the next steps in identifying cancer at a significantly earlier stage as compared to current oncology imaging modalities.

### 3.2. Cardiovascular Imaging

MPI offers benefits that are increasingly attractive for use in cardiovascular imaging [56]. Magnetic nanoparticles can act as nano-probes and deliver significant information including anatomic and physiologic details of cardiovascular diseases [57]. This behavior is largely a result of the size and physical characteristics of MNPs, as they are well suited for cellular imaging of myocardial and atherosclerotic anatomy and abnormalities [38]. A main application of MPI for cardiovascular imaging is direct administration of MNPs into blood vessels to visualize blood flow. This is feasible due to the ability of MPI to image any depth of tissue [13] and is quite impactful, as visualizing blood flow can identify abnormalities in velocity or pattern of blood flow [58], which can indicate structural changes. Vaalma et al. [59] described the ability of MPI to investigate degree of stenosis within vasculature. It was found that at an appropriate clinical MNP concentration, an area of stenosis as small as 2 mm could be successfully imaged. MPI can also be used during cardiovascular interventions, such as catheter guidance in minimally invasive procedures [27].

Current cardiovascular imaging techniques such as PET and MRI have artifacts in images due to long imaging times during data acquisition [56]. MPI presents solutions to this problem as it has a high signal to noise ratio and SPIONs offer a much quicker response time [59]. As a result, MPI for cardiovascular imaging, particularly in combination with modalities such as MRI or CT is an area of particular interest [56,60].

Lu et al. [60] utilized an ultrasmall superparamagnetic iron oxide (USPIO) ferumoxytol as the contrast agent for MRI and targeted cardiovascular imaging. Ferumoxytol is currently clinically available in the US for treatment of iron deficiency anemia and is accompanied by safety concerns. USPIO characterizes pathology in patients by localizing macrophage infiltration, which strengthens T2 imaging and makes this off label usage interesting. Lu et al. [60] also concluded that substantial research is needed to improve the specificity of targeted imaging utilizing ferumoxytol. It is possible that this research can be enhanced by utilization of MPI. Franke et al. [61] utilized SPIONs and MPI in conjunction with MRI to generate high-resolution images of hemodynamic flow in the heart. This combined imaging technique allows non-invasive imaging for cardiovascular assessment to potentially be used for diagnostic imaging in clinical applications. Magnetic nanoparticles are being developed to improve spatial resolution and contrast of cardiovascular images.

Mohtashamdolatshahi et al. [62] synthesized a novel multicore nanoparticle for direct comparison against commercial contrast agent, Resovist. The multicore particles provided significantly higher resolution at a lower dose than Resovist. As a result, the inferior vena cava and abdominal aorta were successfully reconstructed using MPI with the multicore nanoparticles. Tong et al. [56] developed a novel multimodal imaging agent, 5-HT-Fe_3_O_4_-Cy7 nanoparticles (5HFeC NPs) by conjugating 5-hydroxytryptamine (5-HT) and Fe_3_O_4_@PEG-COOH with cyanine 7 N-hydroxysuccinimide ester (Cy7-NHS). This agent was able to be utilized to actively target myeloperoxidase (MPO), which is an inflammatory protein, in a previously established MPO-implanted mouse model. This work was pursued to establish an appropriate multimodal method to image high-risk atherosclerotic plaques. Imaging was accomplished using in vivo fluorescence imaging (FLI) and MPI and compared to MPI and computed tomographic angiography (CTA). Results demonstrated that this specific nanoprobe can be utilized with FLI, MPI and CTA to image active MPO in atherosclerosis with high sensitivity. With additional exploration, this could be used for in vivo monitoring of MPO activity and identification of vulnerable atherosclerotic plaques.

The studies described above highlight the promise that MPI and specific MNPs hold for highly sensitive and specific multimodal imaging of the cardiovascular system. Without doubt, additional work is needed to determine ideal MNPs for cardiovascular imaging but investigations are ongoing and in vivo study results are encouraging.

### 3.3. Neuroimaging

MPI enables and improves many neuroimaging applications and is often considered safe. MPI not only provides superlative sensitivity and accuracy to other imaging modalities, but can also be used anywhere in the body and utilizes long-circulating SPIONs [63] which leads to great promise for neuroimaging. By providing 3D information, MPI is a superior technique over common 2D methods such as X-ray or MR angiography. Traditional X-ray and MR imaging methods are often unreliable in evaluating size and location of brain tumors, and are unable to differentiate the pathophysiology of changes in tumor size [64]. MPI has the potential to image smaller or elusive tumors more specifically, and hopefully improve clinical outcomes through safe and specific diagnosis and monitoring of brain cancer [21].

Received signals from MPI methods are directly related to the volume of blood in the vessel which is a distinct advantage compared to 2D imaging methods. Furthermore, background noises from the surrounding tissues and calcium are not a concern in MPI [21]. Short-term tracking of changes can be achieved by tracing the blood pool with the help from MPI-specific long circulating SPIONs coated with PEG [65]. In a similar study, rapid in vivo detection and quantification of gastrointestinal (GI) bleeding was enabled using PEG-stabilized SPIOs as long circulating tracers where heparin was used as an anticoagulant in order to induce GI bleeding [63]. In brain tumor studies, where the access to undamaged brain tissues is blocked and the tumor has been hypervascularized with leaky vessels, the size of SPIO nanoparticles can be optimized to passively target and accumulate in the tumor [21,51]. Surface modification of SPIOs has been used to enable active tumor targeting, for instance, lactoferrin conjugated SPIO nanoparticles have been used to target brain glioma cells in MPI [66].

There are a few studies focusing on the potential of using functional MPI (fMPI) in neuroimaging [67,68]. fMPI has the potential to strengthen the ability to monitor brain activation or deep brain stimulation by detecting changes in cerebral blood volume (CBV). fMPI would offer advantages over current methods such as PET and functional magnetic resonance imaging (fMRI) by minimizing safety concerns such as radioactive elements, overcoming low imaging precision, [68,69,70] and offering background signal free images [67,70]. Cooley et al. [71] used hypercapnic manipulation in a rat model along with a relatively simple single-sided MP drive coil and detector to demonstrate the ability of MPI to detect functional CBV changes through monitoring of SPION concentration. This work highlighted the possibility of utilizing fMPI for neuroimaging and emphasized that further research is needed to develop a system appropriate for small animal fMPI. This is in line with the current research in fMPI that is focused on designing fMPI scanners and systems that offer high spatial resolution and sensitivity and are appropriate for human imaging [67,68].

**Figure 2:**
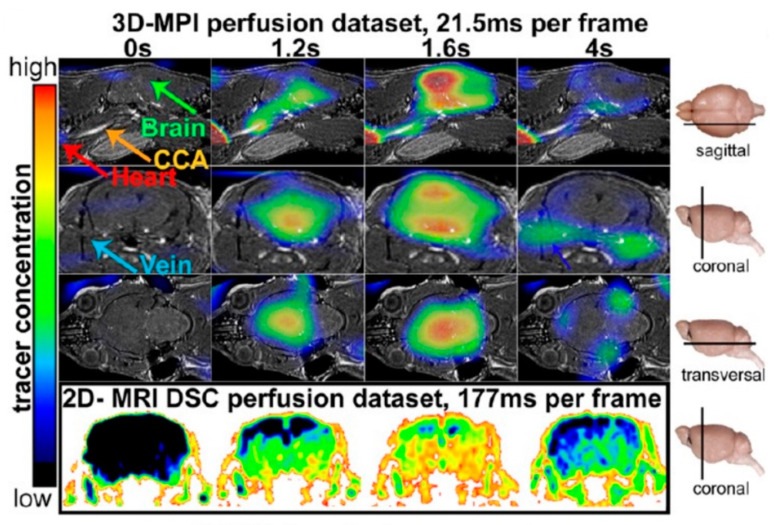
Tracers of SPIO bolus passing through the stroked brain of a mouse. Slices of fused 3D MPI data at several times are shown for the coronal, sagittal, and transverse planes. (Red arrows: occluded and non-occluded common/internal carotid. Blue arrow: the basilar artery). MR dynamic susceptibility contrast (DSC) images are shown for comparison (bottom row). Reprinted with permission from Ludewig et al. [72].

MPI methods are assumed to have shorter acquisition times as well as higher temporal resolution when compared to MRI or CT. Recognition of the anterior and posterior cerebral circulation via the basilar artery has been performed via MPI methods on the mouse brain, where the real-time observation of the flowing blood has been enabled. Additionally, MPI has been used by Ludewig et al. [72] to assess cerebral perfusion in mice. Figure 2 displays signal information obtained from the injected tracers of SPIO at various time points, highlighting a useful technique in diagnosis and therapy of patients affected by stroke.

Further research and development is undoubtedly needed in the field of neuroimaging and fMPI, particularly in designing an MPI scanner appropriate for the size of the human body [20,67,68]. However, the numerous applications of MPI are apparent, and the potential of this imaging modality to change the way physicians diagnose and monitor neural tumors whether benign or malignant, is exciting.

### 3.4. Cell Tracking 

Current areas of interest of MPI for cell tracking include evaluation of cell delivery and fate, particularly for cardiovascular and neural applications [73,74]. Prior to investigation of MPI, techniques for evaluating cell transplantation were based upon post-mortem histopathology, which effectively limits potential for real-time longitudinal monitoring and clinical utility [75,76], as well as imaging modalities including BLI, fluorescence imaging and PET, which are each accompanied by challenges. BLI and fluorescence imaging have been used in vivo, but provide low-resolution 2D images, do not allow linear quantification of cells and do not provide adequate depth penetration [73]. PET and SPECT provide appropriate depth penetration and sensitivity, but expose patients to radiation, and the tracers used have limited half-lives [20,73,76], which creates a delicate balance between safety and utility of these modalities, particularly for longitudinal monitoring.

Prospective applications for MPI include monitoring and therapy of cardiovascular and neural diseases utilizing stem cell therapy, [77,78,79] as well as development of safe, effective stem cell therapies [80]. Benefits of MPI for cell tracking in these areas include high sensitivity and specificity [75], especially the promise of specific quantification of transplanted cells [75,81], long-term longitudinal monitoring of applied cells [76] and perceived risk reduction, as SPIONs utilized are described as biocompatible and biodegradable [75]. SPIONs are among the most employed tracers for labeling stem cells due to their exceptional magnetic properties and outstanding biocompatibility [82,83].

**Figure 3:**
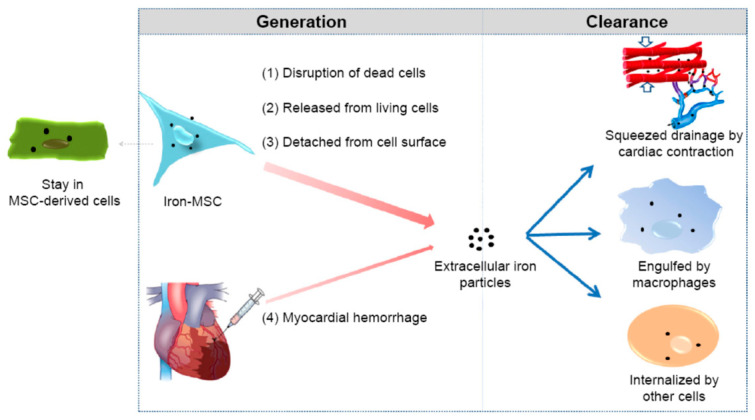
Generation and clearance of the extracellular SPIONs in the myocardium. Reprinted from Huang et al. [90] following the Creative Commons Attribution (CC BY) license (http://creativecommons.org/licenses/by/4.0/) (accessed on 4 March 2021).

MPI has potential to be used with MRI to mark and track stem cells for treatment of cardiovascular diseases, as stem cell therapy is a promising therapeutic option for cardiovascular diseases, including myocardial infarctions [77,78,79,84]. Figure 3 shows a schematic of generation and clearance of the extracellular SPIONs in the myocardium. SPION-MRI methods show promise, as they can be utilized to mark and track stem cells for various time courses, and have the potential to eliminate challenges from short tracer half-lives. Zheng et al. [74] demonstrated the ability to quantitatively track SPIO-labeled human mesenchymal stem cells (hMSCs) administered intravenously in a rat model utilizing MPI and CT. They also exploited the long-lasting nature of the SPIO tracer to demonstrate SPIO tracer clearance from the body through longitudinal MPI signals. Similarly, Wang et al. [85] demonstrated the ability of MPI to quantitatively track pancreatic islet cells labeled with dextran-coated Ferucarbotran SPIOs (VivoTrax, Magnetic Insight Inc., Alameda, CA, USA) in a mouse implantation model. MPI was found to be sensitive and specific, and demonstrated a linear MPI signal increase with the number of labeled islet equivalents, with an R^2^ value of 0.988.

Despite existing successes with SPION-MRI, the specific quantification and tracking of therapeutic stem cells remains challenging [86,87]. Specific challenges of SPION-MRI include poor distinction between viable and non-viable cells [88], which is of great interest during treatment [89], unreliable quantification of SPION concentrations within tissues [1], and creation of “black holes” by contrast agents, which are particularly prominent in hemorrhagic tissues or air-tissue interfaces [1]. Fidler et al. [89] built a magnetic particle spectrometer (MPS) with the goal of developing a system to allow for estimation of cell vitality of MNP labeled hMSCs. This work was performed in in vitro cell culture and noted significant changes in the MPS spectra during cell degradation as induced by sodium dodecyl sulfate (SDS). Changes in MPS spectra can be attributed to a change in Brownian relaxation as the cells dissolve and nanoparticles behave as unbound particles. Authors conclude that effects are dependent upon the individual iron oxide nanoparticles, including the coating, and that it is possible to monitor the viability of hMSC vitality within cell culture. This system was able to perform quantitative, highly sensitive measurements and holds promise for future in vitro and in vivo use in combination with optimized particles.

Current challenges include safety and efficacy in labeling. There are many different methods to label cells with SPIONs, and most provide comparable signal quantitation [1]. It was shown that extracellular SPIONs generate longer lasting signals as compared to intracellular NPs. Higher concentrations of extracellular particles is due to iron extrusion from the injected cells and lack of major iron clearance in the myocardium. This longer-term retention of SPIONs may disturb cell viability due mainly to induction of oxidative stress.

**Figure 4:**
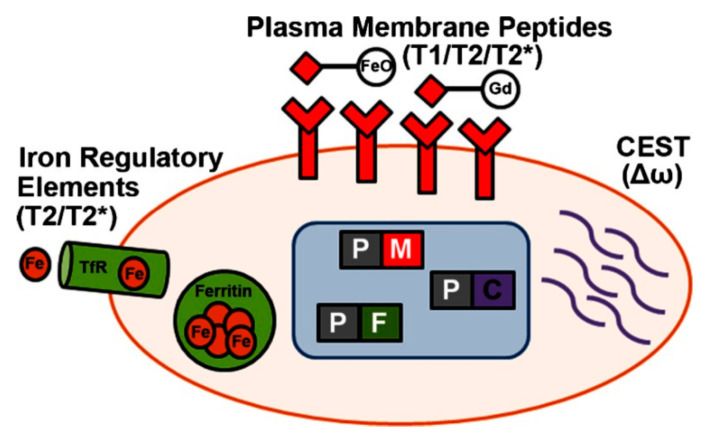
Schematic representation of MRI gene reporter integration for in vivo detection of cell fate decisions. CEST (chemical exchange of saturated magnetization), TfR (transferrin receptor). Reprinted from Vandsburger [92] following the Creative Commons Attribution (CC BY) license (http://creativecommons.org/licenses/by/4.0/) (accessed on 6 March 2021).

Leakage of SPIONs into the adjacent cells is also an obstacle in using these nanoparticles for stem cell tracking and monitoring. Leakage occurs mainly due to exocytosis after cell division [91]. Vandsburger [92] suggested labeling cells with MR reporter genes as a solution to cope with cell tracking modalities. The process is shown schematically in Figure 4.

As highlighted through the studies of Zheng, Fidler and Huang, there have been strides taken in stem cell tracking utilizing MPI. The potential for monitoring cell vitality and to monitor longitudinally is exciting. Currently, further work is required to determine optimal techniques for labeling to minimize iron extrusion and leakage, as well as to create an appropriate MPI scanning system to facilitate safe and sensitive human imaging.

### 3.5. Magnetic Hyperthermia

Magnetic hyperthermia (MH), which is also referred to as nanoparticle based magnetically induced hyperthermia (NP-MIH) [93] and magnetic fluid hyperthermia (MFH) [94], is a technique that utilizes MNPs to couple magnetic energy into the body to ablate diseased tissue [94,95]. Hyperthermic treatment to heat and eliminate diseased tissue, typically cancerous, is not a new concept. It has been described by Milligan in 1984 [96] and reviewed as an adjunctive cancer treatment by Wust et al. in 2002 [97]. Hyperthermic treatments have been found to offer advantages over traditional cancer treatments, and magnetic hyperthermia has been found to offer advantages over non-magnetic hyperthermic treatments [93].

Magnetically induced hyperthermia was shown by Gupta and Sharma [98] to enhance activity of chemotherapeutic agents by increasing permeability of the BBB, which increased drug concentrations within tumors. This increased synergy of magnetic hyperthermia with conventional cancer therapies is well confirmed [13]. Magnetic hyperthermia also offers safety compared to techniques such as whole-body hyperthermia [93]. MH can kill cancer cells without an excessive temperature rise [13]. This is partially a result of the disruption of enzymatic activities within cancer cells, which allows for heat-controlled necrosis [93]. as well as the fact that tumor cells are typically more thermosensitive than healthy, non-neoplastic cells [93]. Despite these strengths, magnetic hyperthermia is accompanied by challenges, including SPION accumulation in off-target organs such as the liver and spleen, which Kut et al. [Kut 2012] demonstrated can result in inadvertent damage to those tissues during heating. An additional limitation lies in focusing of the excitation wave, especially with tissue depth, and difficulty in quantitative imaging of SPION mass for planning of hyperthermia treatment protocols [13].

A strategy to overcome these limitations and strengthen the field of magnetic hyperthermia is the utilization of MPI for image guidance and user-defined spatial localization of magnetic hyperthermia therapy [13,94]. Tay et al. [13] utilized MPI to guide MFH in vivo and found that MPI was able to accurately quantify amounts of iron oxide administered. This could predict the thermal dose to be deposited in the target tissue using a forward model workflow. Murase et al. [99] created a device that allowed for magnetic hyperthermia in the presence of an external static magnetic field (SMF) with an FFP using a Maxwell coil pair. They were able to derive an empirical equation to describe the energy dissipation of MNPs in the presence of alternating and static magnetic fields via phantom experiments. From this work, they found that utilization of an external SMF with a FFP can be effective for controlling the temperature rise in magnetic hyperthermia and therefore reduce the risk of damaging healthy tissue. The combination of magnetic hyperthermia and MPI can offer the ability to understand nanoparticle biodistribution and to non-invasively measure temperature during heating. Hensley et al. [94] reported the first combined MPI-MFH system and were able to demonstrate selective heating of nanoparticle samples within 3 mm of non-user-selected nanoparticles. This work showcases the possibility for MPI and MFH to be combined into one device to provide dual imaging and therapeutic capabilities. Sadhukha et al. [95] investigated inhalational and tracheal instillation as delivery methods for epidermal-growth factor receptor (EGFR)-targeted SPIONs to treat non-small-cell lung cancer (NSCLC) in an orthotopic lung tumor mouse model. Results showed enhanced tumor retention of EGFR-targeted SPIONs with minimized systemic exposure, enhanced intra-tumoral SPION distribution via inhalation, and significant inhibition of in vivo tumor growth. This work suggests the potential of MH as a therapeutic for non-small-cell lung cancer. These capabilities will strengthen research and clinical magnetic hyperthermia and ability to ensure appropriate treatment of tumors [37] and could provide the ability for continuous monitoring of tumors and real quantitation of temperature [94,100].

An intriguing prospective application of magnetic nanoparticles within magnetic hyperthermia is hyperthermia treatment utilizing magnetic biomaterials [101]. MNPs are recognized as promising and suitable heating agents for the design of hybrid materials and implants [102]. Magnetic biomaterials to facilitate hyperthermia therapy have been investigated for local treatment to prevent reoccurrence of resected bone tumors such as osteosarcoma [101,102,103] and for treatment of non-resectable intraluminal tumors such as tracheobronchial carcinoma [102]. Major challenges in this area include the manufacturing of predictable and effective magnetic biomaterials and need for strategies to plan and monitor temperature during treatment [101,103]. Lodi et al. [101] developed a multiphysics model to describe the radiofrequency hyperthermia of residual osteosarcoma cells by using a magnetic implant as a hyperthermia agent. This model is proposed to be used to plan target volume and control heating of tumors while protecting surrounding tissues. During this work, they discovered non-uniformity in distribution of MNPs within the examined scaffolds. This area presents an intriguing potential application for MPI, as it is possible that MPI could be utilized for imaging of implanted magnetic biomaterials and may be able to non-invasively measure temperature during heating.

## 4. Safety and Toxicity

### 4.1. Background

While MPI offers many benefits, there are serious safety concerns. Concerns stem from the magnetic nanoparticles utilized within MPI, as well as the equipment involved. Iron oxide nanoparticles are the most common MNPs studied for this application, and implementation in vivo demands biocompatibility. There is a basic understanding of MNP toxicity. However, further work is required to define specific safety and toxicity parameters. In particular, degradation profiles of available MNPs should be determined, and amounts of potentially toxic substances released from these particles, quantified.

Of all the nanoparticles submitted for evaluation for clinical use, iron oxides remain the most popular among nanoscale medical sciences. Iron oxides possess superparamagnetism, are stable in aqueous solutions and provide appropriate size control and uniformity, low sensitivity to oxidation, promotion of specific interactions when functionalized, and penetration of cell and tissue barriers. These characteristics make iron oxides superior compared to other metal nanoparticles. Regardless of the material, nanoparticle toxicity is dependent upon the size, shape, concentration, dosage, structure, solubility, immunogenicity, pharmacology, and biodistribution [46]. Variations of these factors may lead nanoparticles to exert deleterious effects on the body, such as oxidative stress, embryotoxicity, mutagenicity, genotoxicity, and vascular embolism. These effects may occur as a result of an accumulation of MNPs in the bloodstream, activation of a foreign body response by the immune system, changes in cell morphology, impaired cell signaling, cell differentiation, or damage to the cytoskeleton in response to the MNPs. With this information, it is apparent that biocompatibility is a primary determinant of nanoparticle toxicity [104,105]. Therefore, understanding the circulation, toxicity and biocompatibility of MNPs in the body are necessary steps to determine safety of the nanoparticles.

As mentioned, the equipment utilized for MPI provides an amount of concern. It is crucial that the specific absorption rate (SAR) and magnetostimulation obey certain safety limits. The safety limits of those measurements characterize the optimal scan parameters involved in MPI, including the drive field strength and frequency. These parameters then impact variables such as scanning speed, field of view (FOV), and signal-to-noise ratio in MPI [41]. When these parameters do not meet the safety limits, the drive field can cause peripheral nerve stimulation. Furthermore, the tracers could induce unintentional magnetic hyperthermia, damaging healthy tissue [41]. With MPI as a pre-clinical imaging technique, there have been few tests on human subjects to analyze the safety hazards involved with drive field and magnetostimulation. The upscaling of equipment to accommodate human bodies entails uncertainties with maintaining safety limits, which generates considerable risks. A study performed by Saritas et al. [41] computed magnetostimulation thresholds on a human arm and leg with a full-body MPI scanner. They computed a mean asymptotic threshold of 14.3 mT-pp (peak-to-peak) with a mean chronaxie time of 289 μs, ultimately corresponding to a magnetostimulation threshold of about 15 mT-pp between frequencies of 25 and 50 kHz. This early study offers exception insight into optimizing MPI parameters such as ascertaining the number of FOVs necessary to cover a particular area of interest.

### 4.2. Toxicity Mechanisms

As previously mentioned, accumulation and distribution of MNPs are a main cause for in vivo toxicity. It is well understood that the biodistribution of MNPs depends on the particle size, particle coating, and mass of particles administered [46,104,106,107,108]. In this section, specific examples of these characteristics that affect MNP biodistribution will be highlighted.

Biodistribution of MNPs has been tracked via opsonization, a process where antibodies mark foreign pathogens (in this case MNPs) for elimination by phagocytes [105]. It was determined that opsonized MNPs were removed from the bloodstream within a few minutes and were distributed as follows: 80–90% through the liver, 5–8% through the spleen, and 1–2% via the bone marrow [108]. Additionally, interaction and toxicity of MNPs is highly dependent upon the particle surface. Therefore, investigation into ideal coatings for functional, excretable MNPs is necessary.

**Figure 5:**
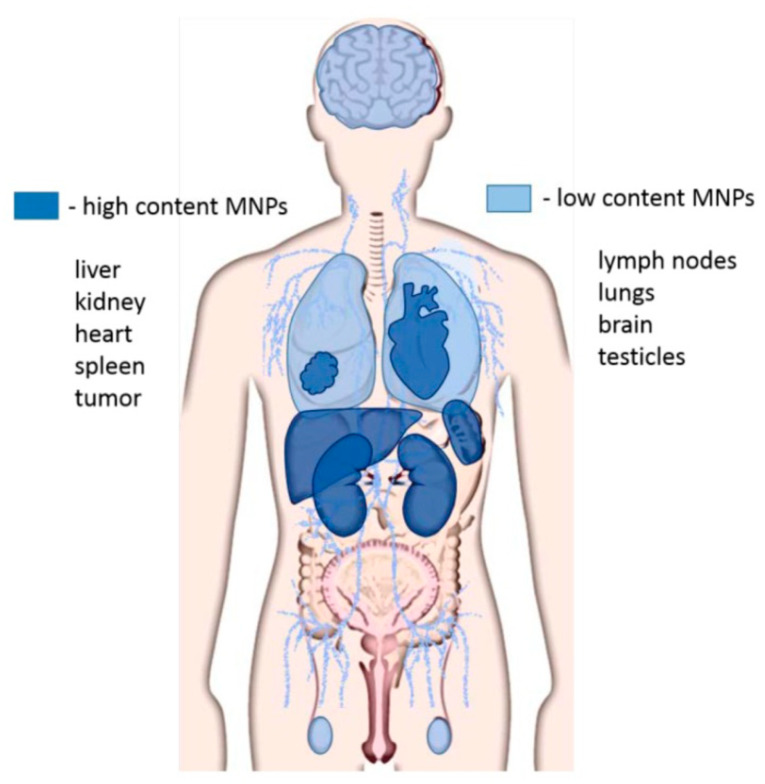
Representation of biodistribution of MNPs in organs and tissues. Unedited image obtained from Zamay et al. [104] following the Creative Commons Attribution (CC BY) license (http://creativecommons.org/licenses/by/4.0/). (accessed on 20 April 2021).

Particle circulation and removal within the body vary depending on particle size and barriers between the circulatory system and tissues. Zamay et al. [104] showed that 40% of MNPs are removed from the endocrine system and excreted through urine 24 h after administration. MNPs were distributed as such: particles with a diameter < 20 nm were excreted by the kidneys, diameters of 30–150 nm were cleared through the bone marrow, heart, kidneys and stomach, and diameters between 150 and 300 nm accumulated within the liver and spleen. Most MNPs accumulate within these organs because they contain numerous macrophages, which are responsible for clearing MNPs and other foreign substances. The distribution of MNPs throughout the body is represented in Figure 5. It is important to note that with uncoated MNPs, a decrease in the size of the particles typically leads to an increase in concentration, resulting in a longer blood circulation time [104].

The most important safety mechanism to be investigated is interference of normal, physiologic iron metabolism after in vivo MNP degradation, as superfluous accumulation of intracellular iron may damage cellular components including nucleic acids and proteins [46]. Therefore, when investigating MNP toxicity profiles, ability to track the accumulation and degradation of these particles through time is crucial. Variation in factors such as the shape, size, structure, and surface of the MNPs will allow elucidation of the most suitable MNP design for specified applications.

### 4.3. Methods for Limiting Toxicity

Desirable properties of MNPs to promote a healthy safety profile mainly include biocompatibility and an appropriate surface chemistry [9]. Of these characteristics, a major technique to effectively reduce toxic effects of these MNPs is surface modification. Surface modifications can: decrease aggregation of MNPs, prevent changes in magnetic properties to maintain efficacy of the MNPs, and inhibit adhesion with plasma proteins to prevent major inflammatory responses in vivo. In particular, increasing the hydrophilicity of nanoparticles reduces the chances of MNPs being detected by macrophages, resulting in longer blood circulation times. This is often desirable in imaging and tracking applications.

**Figure 6:**
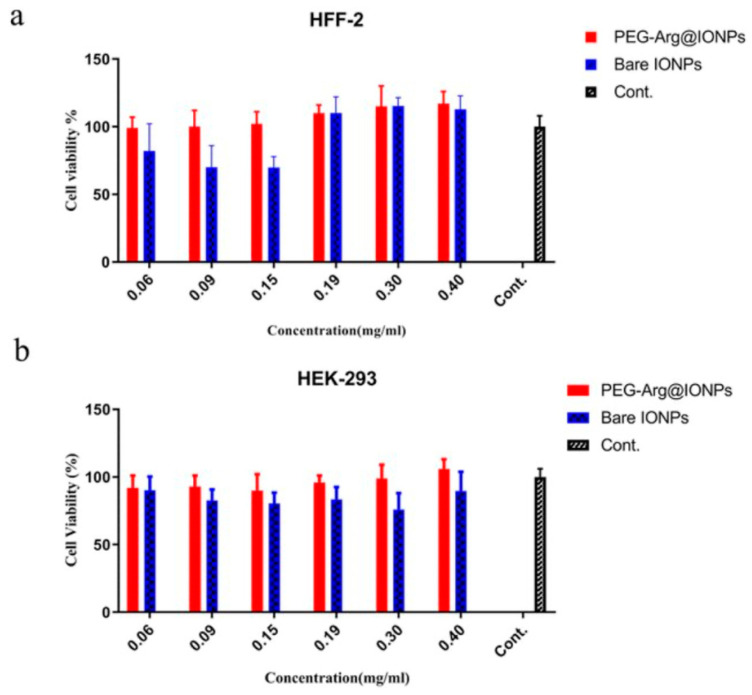
Cytotoxicity analysis of human fibroblast (HFF-2, **a**) and human embryonic kidney (HEK-293, **b**) cell lines with varying concentrations of SPIONs. Unedited image obtained from Nosrati et al. [110] following the Creative Commons License, (http://creativecommons.org/licenses/by/4.0/). (accessed on 20 April 2021).

Peptides, antibodies, polysaccharides, aptamers, and other small acidic molecules can be utilized as target ligands with functional properties which promote these positive surface modifications. Ligands offer MNPs functionalization, which can increase bloodstream circulation time, and facilitate targeted delivery. One study demonstrated that aptamers significantly increased the toxicity threshold, exhibiting no cytotoxicity in vitro nor in vivo, even with high concentrations (100–200 μg/mL) [109]. A study conducted by Nosrati et al. [110] modified the surface of MNPs by capping iron oxide magnetic nanoparticles with arginine, an amino acid, and then conjugating PEG on the surface. Cytotoxicity and hemocompatibility tests were conducted with an in vivo mouse model and an in vitro hemolysis assay, respectively. Specifically, cell viability was tracked through colorimetric assays at different sample concentrations between a fibroblast and kidney cell line (shown in Figure 6). The modified NPs were compared to a group of iron oxide nanoparticles with a bare, unmodified surface.

Results indicated no noticeable effects of the PEG-Arg@IONPs against cell line growth, and less than 2.8% hemolytic activity, demonstrating desirable blood biocompatibility. Lastly, in vivo clearance and biodegradation of MNPs were monitored by MRI techniques and showed long blood circulation time and safe degradation of MNPs [110]. While there are many forthcoming methods to enhance the surface composition of MNPs, this capping method is a relatively new method that resulted in lower cytotoxicity than an unmodified surface. In conclusion, functionalizing MNPs through surface modifications and coatings can increase the biocompatibility and mitigate concerns of accumulation or adverse host responses.

To advance the use of MPI, it is crucial to analyze the average blood circulation time and biodistribution of MNP clearance. With this in mind, it is vital to conduct and document experiments that were successful in minimizing toxicity. A study by Keselman et al. [111] successfully demonstrated extension of MNP blood circulation time and that macrophages and healthy cells remained unharmed for a given period of time. They assessed the in vivo organ biodistribution and clearance time of two tracers, Ferucarbotran (Meito Sangyo Co., Japan) and LS-008 (LodeSpin Labs, Seattle, WA, USA) in female rats. Results showed that most tracers from Ferucarbotran were immediately filtered through the liver while the LS-008 tracers were filtered through the spleen after circulating in the bloodstream for several hours. This study concluded that MPI was able to track both short-term biodistribution and long-term clearance of the tested nanoparticles. Overall, this study highlights the ability of MPI to mitigate toxicity concerns while simultaneously tracking the short-term biodistribution and long-term clearance of two common MPI tracers. This work demonstrates that tailored MNP tracers may be more safely optimized for specific applications.

## 5. Conclusions and Perspectives

As discussed in this review and supported in the literature, MPI is a pre-clinical imaging modality that possesses remarkable promise in enhancing biomedical imaging. MPI offers some distinct advantages compared to current imaging modalities such as MRI, CT, BLI, PET and SPECT. MPI promises to deliver highly sensitive, high-resolution, 3D images without the background tissue signals of MRI, and to eliminate deep tissue imaging concerns regarding depth attenuation and bypass the risk of ionizing radiation. These are limitations seen in BLI and PET/SPECT, respectively. Additionally, MPI has demonstrated strong potential for use in cell tracking and longitudinal monitoring, which could elevate capabilities in the field of regenerative medicine and provide invaluable tools to researchers and one day, clinicians. Despite these benefits, MPI is unlikely to completely replace current imaging modalities, as it is often required to be complemented with an additional modality that can provide anatomic morphology, such as MR or CT.

MPI has not yet become widely accepted and practiced. This is partially a result of lack of available hardware and scanning systems appropriate for the human body. While there are working models that are appropriate for rodents or small animals, the process of scaling an MPI scanner for human use is still underway. Additional limitations include safety concerns surrounding MPI systems, which include risk of PNS and tissue heating, as well as concerns regarding safety and toxicity of MNPs implemented within MPI applications. While new MNPs are being developed to enhance biocompatibility and minimize toxicity concerns, more research is necessary to bring awareness to this application and provide more extensive data to be used in future biomedical applications.

Overall, MPI offers great promise for a wide range of applications, including but not limited to cancer, cardiovascular, pulmonary and neuroimaging, cellular tracking, and therapeutics such as magnetic hyperthermia. Currently, MPI remains a pre-clinical imaging modality and requires significant upscaling and safety profile development prior to being utilized clinically. However, over time and with extensive research and development, the field of magnetic particle imaging will no doubt revolutionize the accuracy, precision, and methodology in which innumerable diseases are diagnosed and monitored, and the therapy that is administered.
